# Safety, tolerability, and efficacy of repeated doses of dihydroartemisinin-piperaquine for prevention and treatment of malaria: a systematic review and meta-analysis

**DOI:** 10.1016/S1473-3099(16)30378-4

**Published:** 2017-02

**Authors:** Julie Gutman, Stephanie Kovacs, Grant Dorsey, Andy Stergachis, Feiko O ter Kuile

**Affiliations:** a Malaria Branch, Division of Parasitic Diseases and Malaria, Centers for Disease Control and Prevention, Atlanta, GA, USA; b Department of Epidemiology, University of Washington, Seattle, WA, USA; c Department of Medicine, San Francisco General Hospital, University of California, San Francisco, CA, USA; d Department of Global Health, University of Washington, Seattle, WA, USA; e Department of Pharmacy, University of Washington, Seattle, WA, USA; f Liverpool School of Tropical Medicine, Liverpool, UK

## Abstract

**Background:**

Intermittent preventive treatment (IPT) for malaria is used in infants, children, adults, and pregnant women. Dihydroartemisinin-piperaquine (DP) is an effective, well tolerated artemisinin-based combination therapy. The long half-life of piperaquine makes it attractive for IPT. We conducted a systematic review and meta-analysis to establish the efficacy and safety of repeated treatment with DP.

**Methods:**

Following PRISMA guidelines, we searched multiple databases on Sept 1, 2016, with the terms: “human” AND “dihydroartemisinin-piperaquine” OR “DHA-PPQ”. Studies were eligible if they were randomised controlled trials (RCTs) or prospective cohort studies involving repeat exposures to standard 3-day courses of DP for either seasonal malaria chemoprevention, mass drug administration, or treatment of clinical malaria, conducted at any time and in any geographic location. Random-effects meta-analysis was used to generate pooled incidence rate ratios and relative risks, or risk differences.

**Findings:**

11 studies were included: two repeat treatment studies (one in children younger than 5 years and one in pregnant women), and nine IPT trials (five in children younger than 5 years, one in schoolchildren, one in adults, two in pregnant women). Comparator interventions included placebo, artemether-lumefantrine, sulfadoxine-pyrimethamine (SP), SP+amodiaquine, SP+piperaquine, SP+chloroquine, and co-trimoxazole. Of 14 628 participants, 3935 received multiple DP courses (2–18). Monthly IPT-DP was associated with an 84% reduction in the incidence of malaria parasitaemia measured by microscopy compared with placebo. Monthly IPT-DP was associated with fewer serious adverse events than placebo, daily co-trimoxazole, or monthly SP. Among 56 IPT-DP recipients (26 children, 30 pregnant women) with cardiac parameters, all QTc intervals were within normal limits, with no significant increase in QTc prolongation with increasing courses of DP.

**Interpretation:**

Monthly DP appears well tolerated and effective for IPT. Additional data are needed in pregnancy and to further explore the cardiac safety with monthly dosing.

**Funding:**

Bill & Melinda Gates Foundation and NIH.

## Introduction

Malaria is a major, preventable cause of morbidity, mortality and adverse birth outcomes in sub-Saharan Africa.^1^, ^2^ Although malaria mortality has fallen as a result of the scale-up of insecticide-treated bed nets and artemisinin-based combination therapies (ACTs), additional efforts are needed.^3^ Intermittent preventive treatment (IPT) of malaria is a strategy for the control of malaria in pregnant women (IPTp), infants, children (seasonal malaria chemoprevention [SMC]),^4^ and potentially in high-risk subgroups of non-pregnant adults and schoolchildren. IPT involves the administration of curative doses of antimalarials at predefined intervals irrespective of malaria infection status.

Of the available ACTs, dihydroartemisinin-piperaquine (DP) is one of the most attractive drugs for IPT. It is effective, with cure rates of 98% or more in non-pregnant and pregnant populations.^5^, ^6^, ^7^ The long half-life of piperaquine (about 23 days [range 19–28] in adults and 14 days [range 10–18] in children)^6^ provides 1–2 weeks' longer post-treatment prophylaxis than artemether-lumefantrine (AL, half-life 3–6 days),^8^ artesunate-amodiaquine (half-life 6–18 days),^9^ or sulfadoxine-pyrimethamine (SP, half-life 4–11 days),^10^ and a similar duration of post-treatment prophylaxis as mefloquine (half-life 10·5–14 days).^11^ It is well tolerated compared with other antimalarials: side-effects are typically limited to minor gastrointestinal adverse events, mild headache, and dizziness.^12^

DP can cause dose-dependent prolongation of the QT interval^13^ and is not recommended in patients with congenital long QT syndrome (about one in 2500 children)^14^ or who are taking other QT prolonging drugs.^13^ Numerous drugs have been associated with QT prolongation, including multiple classes of antibiotics (eg, erythromycin,^15^ quinolones,^15^ co-trimoxazole^16^) and antimalarials.^17^ Mild QT prolongation is clinically silent, but extreme prolongation can cause arrhythmias, including torsade de pointes, a potentially fatal polymorphic ventricular tachycardia occurring in roughly one of 10 000 exposures to QT prolonging drugs.^18^ Diagnosis of prolonged QT requires electrocardiograms (ECG); the normal range differs for men and women, as well as children and adults. Few studies of DP have assessed ECGs.^19^, ^20^

**Research in context**

**Evidence before this study**

Malaria is a major, preventable cause of morbidity, mortality, and adverse birth outcomes in sub-Saharan Africa. Intermittent preventive treatment (IPT) of malaria, which involves curative doses of antimalarials at predefined intervals irrespective of malaria infection status, is a strategy for the control of malaria in pregnant women, infants, and children (seasonal malaria chemoprevention [SMC]). Dihydroartemisinin-piperaquine (DP) is an effective, well tolerated antimalarial, and the long half-life of piperaquine makes DP an attractive choice for IPT. However, DP is known to cause dose-dependent prolongation of the QT interval, and limited data exists on whether the risk of QT prolongation is increased with repeated dosing. We conducted a systematic review and meta-analysis to establish the efficacy and safety of repeated treatment with 3-day courses of DP.

We searched Medline, Embase, Web of Science, Scopus, CINAHL Plus, the Cochrane Library databases, WHO Global Health Library, the Malaria in Pregnancy Consortium (MiPc) Library, ‘grey literature’ databases (unpublished literature including ongoing clinical trials, ongoing PhDs, unpublished PhDs, aborted research, and any other unconventional unpublished literature on the topic), and conference abstracts for articles published before Sept 1, 2016 using the terms: “human” AND “dihydroartemisinin-piperaquine” OR “DHA-PPQ”, and restricting the language to English. There are several reviews on the safety and efficacy of a single course of DP for treatment (ie, case management), and one review of studies of IPT in children (now called SMC) (including two using DP or other piperaquine combinations) and one of IPT in schoolchildren (including one trial using DP).

**Added value of this study**

To our knowledge, this is the first review and meta-analysis to specifically assess the safety and efficacy with repeated courses of DP for case management, IPT, mass drug administration or seasonal malaria chemoprevention in all age groups when compared with placebo or other antimalarial interventions. Monthly DP was more effective than most other options for the prevention of malaria, and appeared to be well tolerated and safe, with less serious adverse events than many comparator interventions and no evidence for increased risk of adverse cardiac events. Nevertheless, data on cardiotoxicity is still scarce.

**Implications of all the available evidence**

DP is a valuable potential candidate for use as IPT and could greatly reduce malaria morbidity and mortality. Additional studies incorporating electrocardiogram measurements are needed to confirm the cardiac safety of repeated monthly dosing.

Administration of DP with food, particularly fat, increases the bioavailability, leading to increased drug concentrations and a greater degree of QT prolongation, which persists for a longer duration.^21^ Additionally, piperaquine concentrations might also be increased when co-administered with drugs that are CYP3A4-inhibitors (eg, some protease inhibitors).^13^ For these reasons, the drug manufacturer recommends obtaining ECGs to monitor therapy when clinically indicated. However, this is not practical if DP is to be given as IPT in resource poor settings and studies assessing the cardiotoxicity of DP when provided for case management show the risk to be low.^22^ Furthermore, neither DP nor AL displayed an in-vitro signal for a significant pro-arrhythmic risk or appear to induce potential torsadogenic effects.^23^ However, piperaquine is eliminated slowly and theoretically this risk might be increased when repeated doses are given, especially when given monthly. We conducted a systematic review and meta-analysis to assess the efficacy, safety, and tolerability of repeated dosing of DP when used for case management, IPT, mass drug administration or seasonal malaria chemoprevention.

## Methods

### Search strategy

We did a systematic literature search according to PRISMA guidelines^24^ on Sept 1, 2016, using simple search terms “human” AND “dihydroartemisinin-piperaquine” OR “DHA-PPQ” (see appendix). Studies were eligible if they were randomised controlled trials (RCTs) or prospective cohort studies involving repeat exposures to standard 3-day courses of DP for either chemoprevention (IPT/SMC), mass drug administration, or treatment of clinical malaria, conducted at any time and in any geographic location. The search was restricted to the English language (appendix).

### Data management

Two independent reviewers (SK, JG) screened titles, abstracts, and full texts and agreed on final study eligibility. Reviewers independently extracted data using a standardised form and database. If required, additional information was obtained from authors.

### Quality assessment

The Cochrane Collaboration's tool was used to assess the quality and risk of bias of clinical trials.^25^ The quality of observational studies was assessed using the Newcastle Ottawa Scale.^26^

### Data analysis

Random-effects meta-analysis was used to generate pooled incidence rate ratios (IRR) and relative risks, or risk differences when there were zero events in both study groups, to compare the effect of DP relative with other antimalarials or placebo on malaria incidence and tolerability; odds ratio (OR) was used for serious adverse events (SAEs) because they are rare events. To correct for studies reporting no SAEs in either the DP or comparison group, a fixed effects model with continuity correction of 0·5 was used to generate Mantel-Haenzel pooled ORs for each study to be informative.^26^

Heterogeneity was expressed as *I*^2^ value and categorised as low if *I*^2^ was 0–40%, moderate if *I*^2^ was 30–60%, substantial if *I*^2^ was 50–90%, and considerable if *I*^2^ was 75–100%.^24^ Analyses were stratified by study type (case management *vs* IPT/SMC/mass drug administration) and geographic location (east Africa, west Africa, and Asia). Due to scarcity of data, we could not stratify on pregnancy status. The influence of study quality on results was assessed by sensitivity analyses. Publication bias was assessed through funnel plots. Two-tailed p-values <0·05 were considered statistically significant.

### Role of the funding source

The sponsor of the study had no role in study design, data collection, data analysis, data interpretation, or writing of the report. The corresponding author had full access to all the data in the study and had final responsibility for the decision to submit for publication.

## Results

**Figure 1:**
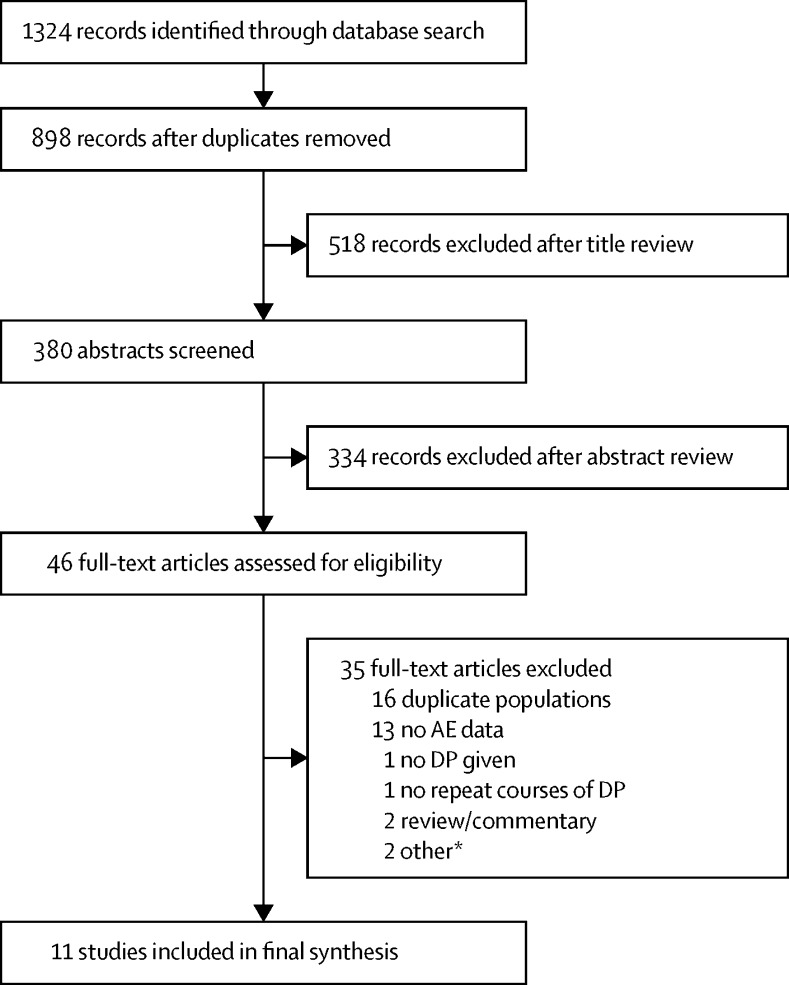
PRISMA flow chart DP=dihydroartemisinin-piperaquine. AL=artemether-lumefantrine. A second trial^29^ reporting on the use of DP for rescue treatment among pregnant women included nine women who received at least two courses of DP (six received three courses and three received two courses), but all women had also received a preceding course of either quinine or intravenous artesunate with or without clindamycin, and there were no control women who had not received DP. *One trial^28^ comparing seasonal malaria chemoprevention (SMC) with sulphadoxine-pyrimethamine plus amodiaquine *vs* placebo SMC (passive case detection and case management with either DP or AL during the malaria transmissions season) was excluded because only 27 of 800 children (3·4%) in placebo SMC group (ie, the DP case management group) received two or more courses of DP and safety data by number of courses received were not available.

Our search identified 898 citations; after title review, 380 abstracts and 46 full text articles (29 distinct studies) were reviewed (figure 1). 11 studies were eligible: one cohort study in pregnant women (n=5288),^30^ one RCT of repeated treatments in children younger than 5 years (n=312),^20^ and nine RCTs with IPT/SMC (henceforth referred to as IPT). Of the nine RCTs, five were in children younger than 5 years (n=5481),^6^, ^31^, ^32^, ^33^, ^34^ one in schoolchildren (n=740),^35^ one in adult men at occupational risk of malaria (n=961),^36^ and two in pregnant women (n=1846;^37^, ^38^ table). In total, there were 14 628 participants; 4883 in DP groups, of whom 4511 were exposed to DP and 3935 received at least two courses of DP, including 762 pregnant women and 1913 children aged less than 5 years. The remaining 9745 were exposed to placebo or other comparator therapy (including 990 exposed to SP–piperaquine). The 4511 participants exposed to DP received a total of 18  873 courses, with 18 297 courses taken by the 3935 participants who received at least two doses, some of whom received as many as 18 monthly doses. Several different dosing intervals were studied, including monthly (including in pregnancy), every 2 months, quarterly, and three times during the second and third trimester of pregnancy. Comparator interventions included placebo, AL, SP, SP+amodiaquine, SP + piperaquine, SP + chloroquine, and piperaquine + co-trimoxazole. All studies were conducted in areas with no or low parasite resistance to piperaquine or the artemisinins.

The Cochrane Collaboration tool scored four RCTs as low risk of bias and six as moderate risk of bias (appendix). The Newcastle Ottawa Scale suggested a moderate risk of bias for the single cohort study.

### Protective efficacy

Repeated first-line course of DP for case management was associated with a 16% lower risk of parasitological treatment failure by day 28 compared with AL, but only one trial provided data for analysis (IRR 0·84 95% CI 0·81–0·86).^20^

**Figure 2:**
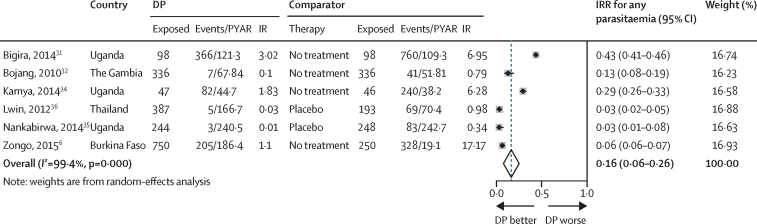
Pooled incidence rate ratio for any parasitaemia, monthly dihydroartemisinin-piperaquine *vs* placebo DP=dihydroartemisinin-piperaquine. PYAR=person-years at risk. IR=incidence rate. IRR=incidence rate ratio. Lwin and colleagues^36^ and Zongo and colleagues^6^ did not report PYAR, instead they reported cumulative incidence over a year. PYAR was calculated based on the incidence rate and number of events. Zongo and colleagues'^6^ numbers are based on intent to treat.

Monthly DP for IPT was associated with an 84% reduction in the incidence of malaria parasitaemia measured by microscopy compared with placebo (pooled IRR; figure 2). This was 75% in east Africa, 91% in west Africa, and 97% in adults in Thailand^36^ (appendix).

**Figure 3:**
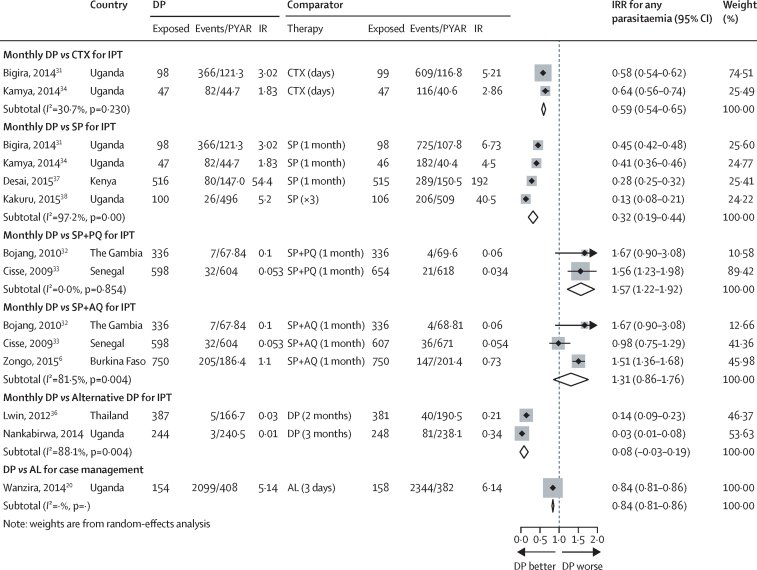
Pooled incidence rate ratio or relative risk for any parasitaemia, monthly dihydroartemisinin-piperaquine *vs* any other therapy DP=dihydroartemisinin-piperaquine. PYAR=person-years at risk. IR=incidence rate. IRR=incidence rate ratio. CTX=co-trimoxazole. SP=sulfadoxine-pyrimethamine. SP+PQ=sulfadoxine-pyrimethamine piperaquine. SP+AQ=sulfadoxine-pyrimethamine amodiaquine. AL=artemether lumefantrine. Lwin and colleagues^36^ and Zongo and colleagues^6^ did not report PYAR. PYAR was calculated based on the incidence rate and number of events. Cisse and colleagues^32^ reported cumulative incidence. Kakuru^37^ reported detection of malaria parasites by LAMP at each visit as the prevalence of positive tests during pregnancy out of all tests. Zongo and colleagues'^6^ numbers are based on intention to treat.

Monthly IPT with DP provided similar efficacy to monthly SP+amodiaquine for preventing any parasitaemia, but inferior efficacy compared with monthly SP+primaquine (figure 3). Monthly IPT-DP was significantly better than daily co-trimoxazole, or monthly IPT-SP for the prevention of malaria infection.

Dosing of IPT-DP on a less than monthly schedule (every 2 months^36^ or 3 months^35^) provided significantly less protection against any parasitaemia than monthly dosing (figure 3).

The considerable heterogeneity (*I*^2^>75%) among placebo controlled RCTs was partly explained by difference in quality of the trials as established by the Cochrane Collaboration's tool:^25^ there was no heterogeneity in the two RCTs classified as having low potential for bias (*I*^2^=0%) but high heterogeneity (*I*^2^=99·6%) among the four RCTs classified as having moderate potential for bias (appendix). Absence of variability in study quality within each comparator drug subgroup precluded further assessment of the influence of the risk of bias on the heterogeneity by comparator drug. Geographic stratification did not explain the heterogeneity (appendix).

Among 3960 participants, after excluding arms of studies where most had received no or only 1 course of DP,^30^, ^37^ 133 SAEs were reported, including 23 in patients receiving DP+co-trimoxazole. Including all 4883 participants in DP groups (3935 of whom received at least two courses), 233 SAEs were reported. An additional four SAEs were reported in 990 recipients of SP+piperaquine. Among 3180 participants receiving other treatments and 5575 receiving placebo, 287 and 186 SAEs were reported, respectively (table 1).

**Figure 4:**
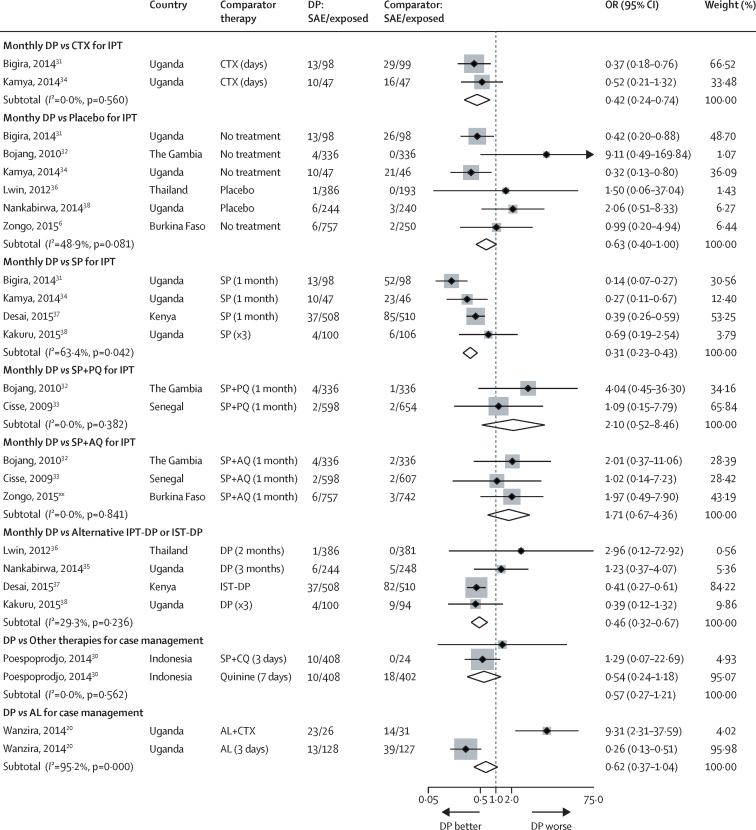
Pooled odds ratios for any serious adverse event after exposure to dihydroartemisinin-piperaquine stratified by comparator therapy DP=dihydroartemisinin-piperaquine. SAE=serious adverse event. CTX=co-trimoxazole. IPT=intermittent preventive treatment. IST=intermittent screening and treatment. SP=sulfadoxine-pyrimethamine. SP+PQ=sulfadoxine-pyrimethamine piperaquine. SP+AQ=sulfadoxine-pyrimethamine amodiaquine. SP+CQ=sulfadoxine-pyrimethamine chloroquine. AL=artemether-lumefantrine. Zongo and colleagues'^6^ numbers are based on actual drug exposures. Poespoprodjo and colleagues^30^: only 64 of 408 DP recipients received two or more courses of DP, but information of SAEs by number of courses received was not available.

After correction for zero events, repeated DP exposure was associated with a significantly lower odds of SAEs compared with placebo, co-trimoxazole, or IPT-SP (figure 4). IPT-DP was also associated with fewer hospital admissions than IPT-SP (appendix). Repeated case management with DP was associated with fewer hospital admissions compared with AL (appendix).

**Figure 5:**
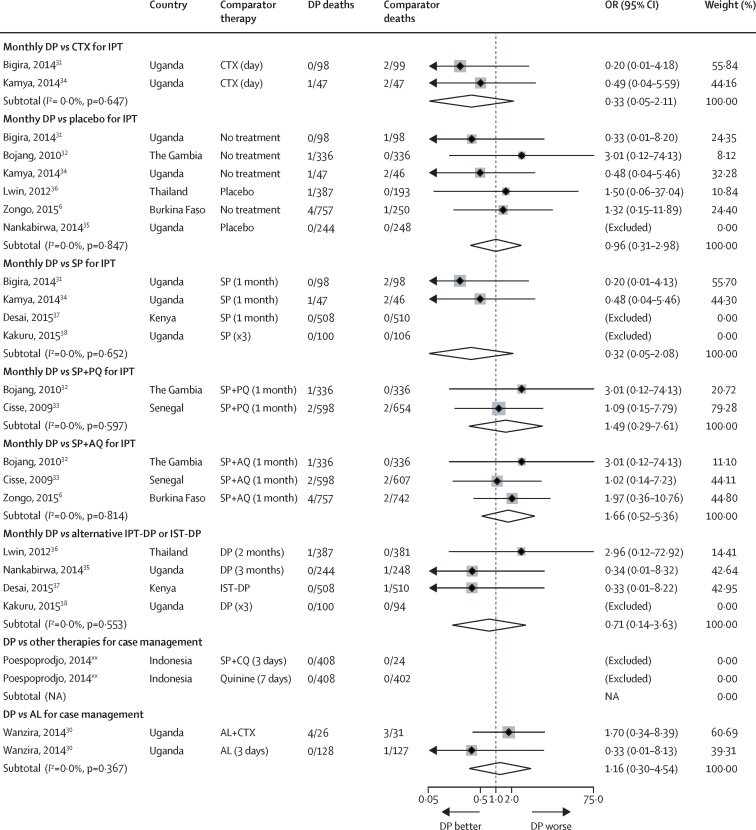
Pooled odds ratios for death after exposure to repeated courses of dihydroartemisinin-piperaquine stratified by comparator therapy Comparisons with zero events in both groups were excluded from the analysis of the pooled OR. OR=odds ratio. DP=dihydroartemisinin-piperaquine. CTX=co-trimoxazole. IPT=intermittent preventive treatment. IST=intermittent screening and treatment. SP=sulfadoxine-pyrimethamine. SP+PQ=sulfadoxine-pyrimethamine piperaquine. SP+AQ=sulfadoxine-pyrimethamine amodiaquine. SP+CQ=sulfadoxine-pyrimethamine chloroquine. AL=artemether-lumefantrine. Zongo and colleagues'^6^ numbers are based on actual drug exposures. Poespoprodjo and colleagues: only 64 of 408 DP recipients received two or more courses of DP.

None of the 11 studies reported SAEs was consistent with sudden cardiac death. Overall, 15 deaths were reported among those exposed to DP, two among those exposed to SP+piperaquine, 18 among those exposed to other comparator therapies, and four among those in placebo groups. No studies reported any sudden or unexplained deaths (figure 5, appendix). IPTp-DP was not associated with an increased risk of loss to follow-up (which could reflect undetected or unreported sudden death) compared with co-trimoxazole, SP, SP + piperaquine, or SP+amodiaquine, but was associated with a 47% higher risk of loss to follow-up compared with placebo (appendix).

The effect of DP on cardiac repolarisation was assessed in 19 HIV-unexposed^31^ and seven HIV-exposed children (Dorsey, unpublished) and 30 pregnant women.^38^ In the 26 children, 183 ECGs were conducted at baseline and follow-up (4–6 h after the third dose of DP with each monthly course); all of the baseline and follow-up ECGs had a QTc less than 450 ms with a mean QTc of 396 ms (SD 31·3, range 278–444). There were no differences in the mean QTc intervals measured after the third dose for children who had been prescribed three to five previous courses of DP (mean QTc 405 ms, SD 26), six to ten previous courses of DP (388 ms, 33), or 11–18 previous courses of DP (396 ms, 33). None of the 30 pregnant women who underwent ECG measurements at 28 weeks' gestational age pre-dosing and post-dosing had QTc intervals greater than 450 ms.^38^ The median increase in QTc from baseline to 4–6 hours after the third dose was 30 ms (range −30 to 50) and 20 ms (−10 to 50) in women randomised to receive monthly DP (n=13) and 3 doses of DP (n=17), respectively, compared with 5 ms (−40 to 60) in women who received three doses of SP (n=12, p=0·57 and 0·28 for monthly and three dose DP compared with three dose SP).

IPT-DP was associated with similar cumulative risk of any vomiting compared with placebo, SP, and SP+amodiaquine, and with a lower risk compared with SP+primaquine (appendix). In the single treatment study with tolerability data, DP was associated with a lower risk of vomiting compared with AL (RR 0·52, 95% CI 0·45–0·61).^20^

In children under 5 years of age, both IPT-DP and IPT-SP were associated with more vomiting during the first course than subsequent monthly courses (DP around 4% *vs* <2%; SP around 3·5% *vs* <2%).^6^ No vomiting was reported among school-aged (6–14 years) children receiving monthly IPT-DP after any of the three courses.^35^ Treatment of clinical malaria with DP was not associated with more vomiting than AL for the first and second courses, and for the third course, participants given DP vomited less than those given AL (2·8% *vs* 7·8% p=0·08).^20^

IPT-DP was not associated with an increased risk of diarrhoea compared with placebo, SP+amodiaquine, SP+primaquine, or IPT-SP in six studies (appendix).

Only four studies provided data on rash or allergic reactions, and no study reported any SAEs due to allergic reactions. IPT-DP was not associated with an increased risk of rash compared with placebo, SP+primaquine or SP+amodiaquine (appendix).

## Discussion

This meta-analysis suggests that DP is as safe as other combinations assessed for IPT or the repeat treatment of clinical malaria, and that it was well tolerated. DP provided superior protection against malaria and resulted in fewer hospital admissions than comparators. In comparison with dosing every 2 or 3 months, monthly administration of DP provided much better protection from malaria, without increasing the risk of adverse events or adversely affecting tolerability.^35^, ^36^, ^37^, ^38^

DP, like some other antimalarials, has been associated with dose dependent risk for QTc prolongation. A previous review assessing the risk of QTc prolongation following a single course of treatment found no difference in the risk for prolonged QTc between DP and AL, but DP was associated with more frequent prolongation of the QTc interval compared with mefloquine-artesunate.^39^ No cardiac arrhythmias or sudden death were reported for any of the drugs, although it is possible that sudden death due to a cardiac arrhythmia could have been incorrectly attributed to other causes. Similarly, in our meta-analysis, no cardiac events were reported among 3935 recipients of repeat courses of DP involving 18 297 courses of DP ranging from two to 18 courses per individual. As only three studies in different populations assessed the effect of DP on the ECG, it was not possible to do a meta-analysis to assess the risk of repeated courses of DP on the ECG; however, no significant QT prolongation was reported with repeat dosing in the individual studies. Furthermore, the risk of death was not significantly increased following receipt of repeat courses of DP, suggesting no significant increased risk of sudden cardiac death, although the rare nature of this event makes it difficult to rule out. It should be noted, however, that although DP was not associated with increased loss to follow-up compared with comparators, there was more loss to follow-up among participants in the DP group in the studies comparing DP against placebo. This was driven primarily by the high loss to follow-up in Lwin and colleagues,^36^ which was unrelated to the intervention since only four withdrew due to adverse events: two from the IPT group and two from the placebo group. The rest were lost due to other reasons.

The clinical relevance of the dose dependent risk for QTc prolongation with DP is not clear, since the pro-arrhythmic potential of piperaquine in vitro appears lower than chloroquine and similar to AL.^23^ One post-marketing study, comparing a compressed 2-day regimen of DP with placebo, has reported clinically significant QT prolongation among participants exposed to DP.^19^ Given the potential for dose accumulation with monthly dosing,^36^ it was reassuring to find that the studies in children did not find evidence that repeat monthly courses were associated with greater degrees of QT prolongation than the first course, even among children that had received ten or more monthly courses^31^ (Dorsey, unpublished data).

It is possible that the absence of additional QTc prolongation with repeat courses reflects the finding that QTc interval returns to normal within approximately 12–48 h following the last dose after each course. Nevertheless, some increase in QTc prolongation with increasing number of courses could not be excluded in pregnant women,^38^ and the strength of the evidence to date is limited because ECGs were only done in three studies involving 56 participants receiving monthly courses, thus more studies are needed. Advances in mobile adapted technology, such as Smartheart or AliveCor, might allow for improved monitoring of patients in remote and resource poor settings in the future.

Our search did not find any studies where repeat courses of DP were provided as part of malaria elimination campaigns that often involve multiple rounds of mass drug administration within a single year. However, our findings with DP as IPT are likely to be generalisable to mass drug administration since both strategies involve asymptomatic carriers of malaria parasites and individuals without malaria parasites at the time of drug administration.

The few people exposed to multiple courses of DP to date precludes our ability to detect an increased risk of an arrhythmia such as torsadogenic event that occur in about one in 10 000 exposures to QT prolonging drugs; our overall sample size can only exclude (95% CI) such events in one in 6099 exposures. The fact that different patient populations were grouped is a potential weakness, as the QTc (and risk for cardiotoxicity) is affected by age, sex, and pregnancy status, and achieved drug concentrations might also vary by patient population and gestational age; however, the paucity of data precluded reviewing the groups individually. The included studies involving young children were conducted before WHO's dose increase for DP in children aged 1–4 years,^4^ and continued collection of safety data with the new dose is needed. One of the treatment studies included many participants who only received a single course of DP. However, no details were provided in the source study that allowed a breakdown of SAEs by number of courses. Finally, it is possible that restricting to English language excluded relevant studies published in other languages.

In this meta-analysis of nearly 4000 patients exposed to repeated courses of DP, IPT-DP was highly effective for the prevention of malaria and reduced all-cause hospital admission compared with other drugs, particularly when provided as monthly courses. Overall, DP was well tolerated, with no evidence of increased frequency of mild or serious adverse events with repeated dosing. The data do not suggest that the known risk of QT prolongation increases with repeated monthly courses, or an increased risk of cardiac events or death following repeated dosing. DP is a valuable potential candidate for use as IPT and ongoing monitoring for cardiac events is needed to provide further reassurance of its safety with repeat doses.

**This online publication has been corrected. The corrected version first appeared at thelancet.com/infection on January 5, 2017**

## Figures and Tables

**Table:** Details of included studies

	**Country**	**Study type**	**Study population**	Comparators*	**Efficacy data**	**SAE**†	**Deaths**	**DOT,**‡**number of courses**
Bigira, 2014^31^	Uganda	Clinical trial-IPT	Children under 5 years including HIV exposed infants	DP 98 SP 98 CTX 99 No treatment 98	Monthly active detection of parasitaemia from 6–24 months of age	DP 13 SP 52 CTX 29 No treatment 26	DP 0 SP 2 CTX 2 No treatment 1	First dose DOT, 1592 courses administered§
Bojang, 2010^32^	The Gambia	Clinical trial-IPT	Children under 5 years	DP 336 (335, 328) SP+AQ 336 SP+PQ 336 No treatment 286	Any malaria within 16-week rainy season (passive surveillance), active detection at study end	DP 4 SP+AQ 2 SP+PQ 1 No treatment 0	DP 1 SP+AQ 0 SP+PQ 0 No treatment 0	All doses DOT, 952 courses administered
Cisse, 2009^33^	Senegal	Clinical trial-IPT	Children under 5 years	DP 598 (578, 539) SP+AQ 607 SP+PQ 654	Passive detection of malaria during 4-month rainy season, active detection at study end	DP 2 SP+AQ 2 SP+PQ 2	DP 2 SP+AQ 2 SP+PQ 2	First dose DOT, 1544 courses administered
Desai, 2015^37^	Kenya	Clinical trial-IPT	Pregnant women in second or third trimester	IPT-DP 516 (516, 477) IST-DP 515 (167, 27) IPT-SP 515	Active detection of parasitaemia at each antenatal clinic visit during pregnancy	IPT-DP 37 IST-DP 82 IPT-SP 85	IPT-DP 0 IST-DP 1 IPT-SP 2	First dose DOT, 1585 courses administered
Kakuru,2015^38^	Uganda	Clinical trial-IPT	Pregnant women in second or third trimester	DP monthly 100 DP ×3 94 SP ×3 106	Monthly assessment with LAMP¶	DP monthly 4 DP ×3 9 SP ×3 6	DP monthly 0 DP ×3 0 SP ×3 0	First dose DOT, 1136 courses administered
Kamya,2014^34^	Uganda	Clinical trial-IPT	Children under 5 years	DP 47 SP 46 CTX 47 No treatment 46	Monthly active detection of parasitaemia from age 4–5 months until age 24 months	DP 10 SP 23 CTX 16 No treatment 21	DP 1 SP 2 CTX 2 No treatment 2	No DOT, drug intake recorded by parents, 561 courses administered§
Lwin,2012^36^	Thailand	Clinical trial-IPT	Adults	DP 387 DP Q2month 381 Placebo 193	Monthly active detection of parasitaemia for 36 weeks	DP 1 DP Q2 month 0 Placebo 0	DP 1 DP Q2 month 0 Placebo 0	All doses DOT, 4089 courses administered§
Nankabirwa, 2014^35^	Uganda	Clinical trial-IPT	School-age children (aged 6–14 years)	DP 244 DP quarterly 248 Placebo 248	Monthly active detection of parasitaemia for 12 months	DP 6 DP quarterly 5 Placebo 2	DP 0 DP quarterly 1 Placebo 0	All doses DOT, 2648 courses administered
Poespoprodjo, 2014^30^	Indonesia	Cohort study-treatment	Pregnant women in second or third trimester	DP 408 (408, 64) ‖ SP+CQ 24 Quinine 402 No treatment 4454	No	DP 10 SP+CQ 0 Quinine 18 No treatment 134	DP 0 SP+CQ 0 Quinine 0 No treatment 0	First dose DOT, 486 courses administered
Wanzira, 2014^20^	Uganda	Clinical trial-treatment	Children under 5 years including HIV exposed infants	DP (+/− CTX) 154 (154, 147)** AL (+/− CTX) 158	Passive detection of parasitaemia before age 5 years	DP 13 DP+CTX 23 AL 39 AL+CTX 14	DP 0 DP+CTX 4 AL 1 AL+CTX 3	First dose DOT, 2218 courses administered
Zongo, 2015^6^	Burkina Faso	Clinical trial-IPT	Children under 5 years	DP 750 (757)†† SP+AQ 749 (742)†† No treatment 250	Monthly active detection of parasitaemia for 4 months	DP 6 SP+AQ 3 No treatment 2	DP 4 SP+AQ 2 No treatment 1	All doses DOT, 2063 courses administered

SAE=serious adverse event. DOT=directly observed therapy. IPT=intermittent preventive treatment. IST=intermittent screening and treatment. DP=dihydroartemisinin-piperaquine. SP=sulfadoxine pyrimethamine. CTX=co-trimoxazole. AQ=amodiaquine, PQ=piperaquine. CQ=chloroquine. AL=artemether-lumefantrine. Q2month=every other month. CHW=community health worker. LAMP=loop-mediated isothermal amplification.

* Numbers in brackets represent the number who received one or more and two or more courses of DP, if reported to be different from the overall sample size in the DP group.

† In addition to any other SAEs reported by the study, all hospital admissions and deaths were considered SAEs. SAEs were reported unrelated to study drugs unless otherwise noted: Bigira and colleagues reported 19 (4·5%) grade 3–4 AEs as possibly related to study drugs, with no significant differences between the intervention groups. Desai and colleagues reported one drug related SAE (an allergic reaction to DP); Kakuru and colleagues reported one patient who developed anaemia after both the first and second dose of DP, after which DP was stopped; Kamya and colleagues reported eight (5·6%) AEs possibly related to study drugs, with no significant differences between the intervention groups; Lwin and colleagues reported that four patients withdrew due to drug related AEs (two in the DP every other month group and two in placebo group).

‡ DOT by study staff, first dose=only the first dose of each course was administered as DOT.

§ The total number of doses was divided by three to estimate the number of courses and rounded to the nearest whole number.

¶ Reported prevalence over the course of pregnancy (incidence was not reported).

‖ Average duration between courses 4·2 months

** Average duration between courses 2·2 months

†† Intention to treat included 750 in the DP group and 740 in the SP+AQ group, but due to allocation errors, 757 were given DP and 742 were given SP+AQ.
