# Supplementary material for: Safety, tolerability, and efficacy of repeated doses of dihydroartemisinin-piperaquine for prevention and treatment of malaria: a systematic review and meta-analysis
Source: Lancet Infect Dis. 2017 Feb;17(2):184–93. doi: 10.1016/S1473-3099(16)30378-4 (PMC5266794; doi:10.1016/S1473-3099(16)30378-4)
Supplement: Supplementary appendix [file mmc1.pdf]

THE LANCET Infectious Diseases

Supplementary webappendix

This webappendix formed part of the original submission and has been peer reviewed. We post it as supplied by the authors.

Supplement to: Gutman J, Kovacs S, Dorsey G, Stergachis A, ter Kuile FO. Safety, tolerability, and efficacy of repeated doses of dihydroartemisinin-piperaquine for prevention and treatment of malaria: a systematic review and meta-analysis. *Lancet Infect Dis* 2016; published online Nov 16. http://dx.doi.org/10.1016/S1473-3099(16)30378-4.

The safety, tolerability and efficacy of repeated doses of dihydroartemisinin-piperaquine for the prevention and treatment of malaria: A systematic review and meta-analysis

Supplementary Web Appendix

Julie Gutman, MD, Stephanie Kovacs, PhD, Professor Grant Dorsey, MD PhD, Professor Andy Stergachis, PhD, Professor Feiko O. ter Kuile, MD PhD

Table of Contents

Supplemental Text ... 2

Supplemental References ... 2

Table S1. PICOTS framework ... 3

Table S2. PubMed search strategy ... 3

Figure S1: Bias assessment of randomized-controlled trials using the Cochrane Collaboration tool ... 4

Figure S2: Pooled incidence rate ratio for any parasitemia, monthly dihydroartemisinin-piperaquine versus placebo stratified by bias assessment ... 5

Figure S3: Pooled incidence rate ratio for any parasitemia, monthly dihydroartemisinin-piperaquine versus placebo stratified by geography ... 6

Figure S4: Pooled odds ratio for any hospitalization, dihydroartemisinin-piperaquine compared to other drugs and placebo ... 7

Figure S5: Pooled risk difference for death with dihydroartemisinin-piperaquine versus comparators 8

Figure S6: Pooled relative risk for loss to follow-up for dihydroartemisinin-piperaquine versus comparators ... 9

Figure S7: Pooled relative risk for vomiting after receiving a dose of dihydroartemisinin-piperaquine compared to other drugs and placebo ... 10

Figure S8: Pooled relative risk for any diarrhea, dihydroartemisinin-piperaquine versus comparator therapies ... 11

Figure S9: Pooled relative risk for any rash, dihydroartemisinin-piperaquine versus comparator therapies ... 12

Supplemental Text

We conducted our search, analyses, and reporting adhering to the PRISMA guidelines for systematic reviews and meta-analyses.[1] An electronic literature search applying the PICOTS framework was conducted of the following clinical databases: MEDLINE, EMBASE, Web of Science, Scopus, CINAHL Plus, the Cochrane Library databases, WHO Global Health Library and the Malaria in Pregnancy Consortium (MiPc) Library.[2] A multi-concept Boolean search strategy was applied using keywords and MeSH terms. We additionally searched 'gray literature' databases, conference abstracts, manually reviewed reference lists of selected publications as well as records recommended by contacting experts so as to encompass a broad range of available literature. We imported all into EndNote Web (Thompson Reuters, NY), removed duplicates, and screened each record against the eligibility criteria.

Data on the study population, including age, severity of malaria, drug exposures, treatment outcomes (including protective efficacy and the incidence of any parasitemia after treatment with DP), tolerability, and all serious adverse events were abstracted. We also used measures of lost to follow-up, drop-outs and adherence as surrogates of tolerability.

Supplemental References

1. Liberati A, Altman DG, Tetzlaff J, et al. The PRISMA statement for reporting systematic reviews and meta-analyses of studies that evaluate healthcare interventions: explanation and elaboration. *BMJ* 2009; **339**: b2700.

2. van Eijk AM, Hill J, Povall S, Reynolds A, Wong H, Ter Kuile FO. The Malaria in Pregnancy Library: a bibliometric review. *Mal J* 2012; **11**: 362.

Table S1. PICOTS framework

Components	Characteristics
**Population**	All persons at risk for malaria or with malaria infections<br>-Subgroup analyses:<br>• Malaria transmission intensity<br>• Geography (Southeast Asia, Sub-Saharan Africa)<br>• Target groups (infants, children, pregnant women, and adults)<br>• Number of doses
**Intervention**	Exposed to repeat DP for treatment or prevention of malaria
**Control**	Exposed to another ACT or antimalarial for treatment, to SP for prevention, or placebo
**Outcomes**	Serious Adverse Events including but not limited to:<br>1. Death<br>2. Any event leading to hospitalization<br>3. QT prolongation<br>4. Adverse pregnancy outcomes (stillbirth, miscarriage, congenital anomalies)<br>Tolerability:<br>1. Vomiting<br>2. Nausea<br>3. Dizziness<br>4. Lost to follow-up, drop-outs or poor adherence
**Timing**	No time limits will be placed on the search
**Setting**	Any study in which participants were exposed to DP including case series<br>Limit to English Language

Table S2. PubMed search strategy

Search date Sept 1, 2016

	Framework	Search terms	Number of articles
**P**	**Population**	Human	
**I**	**Intervention**	AND<br>Dihydroartemisinin piperaquine OR DHA-PPQ	**P + I: 252**
**C**	**Control**	-	
**O**	**Outcome**	-	
**T**	**Timing**	-	
**S**	**Setting**<br>**Limit to English language**	AND (English [la])	**P+I+O+T+S: 244**

Figure S1: Bias assessment of randomized-controlled trials using the Cochrane Collaboration tool

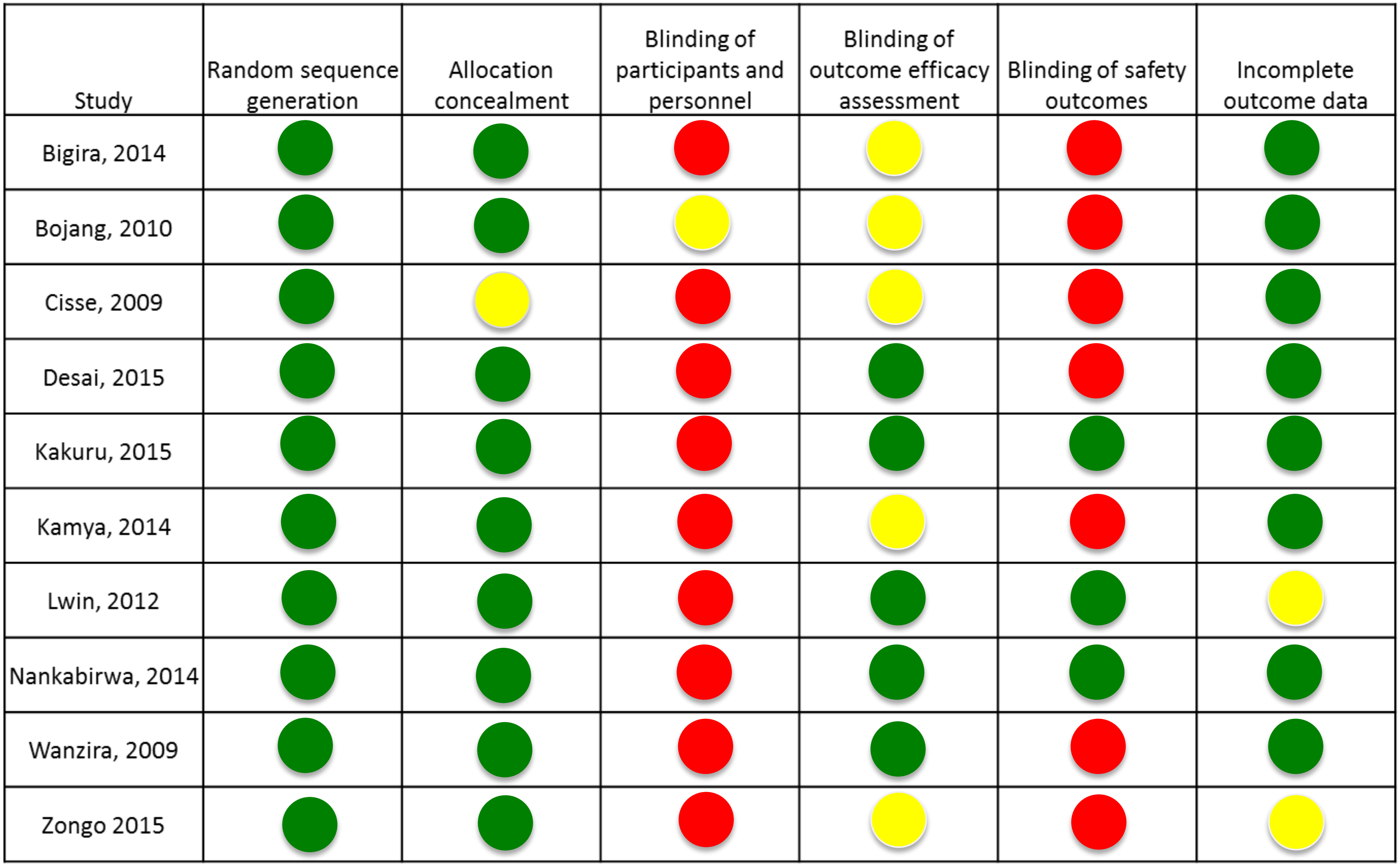

Figure S2: Pooled incidence rate ratio for any parasitemia, monthly dihydroartemisinin-piperaquine versus placebo stratified by bias assessment

Author, Year	Country	DP exposed	DP Events/PYAR*	DP IR	Comparator therapy	Comparator exposed	Comparator Events/PYAR	Comparator IR	IRR for any parasitemia (95% CI)	% Weight
moderate										
Bigira, 2014	Uganda	98	366/121.3	3.02	No treatment	98	760/109.3	6.95	0.43 (0.41, 0.46)	16.74
Bojang, 2010	The Gambia	336	7/67.84	.1	No treatment	336	41/51.81	.79	0.13 (0.08, 0.19)	16.23
Kamya, 2014	Uganda	47	82/44.7	1.83	No treatment	46	240/38.2	6.28	0.29 (0.26, 0.33)	16.58
Zongo, 2015	Burkina Faso	750	205/186.4	1.1	No treatment	250	328/19.1	17.17	0.06 (0.06, 0.07)	16.93
Subtotal (I-squared = 99.6%, p = 0.000)									0.23 (0.02, 0.44)	66.48
.										
low										
Lwin, 2012	Thailand	387	5/166.7	.03	Placebo	193	69/70.4	.98	0.03 (0.02, 0.05)	16.88
Nankabirwa, 2014	Uganda	244	3/240.5	.01	Placebo	248	83/242.7	.34	0.03 (0.01, 0.08)	16.63
Subtotal (I-squared = 0.0%, p = 0.949)									0.03 (0.02, 0.04)	33.52
.										
Overall (I-squared = 99.4%, p = 0.000)									0.16 (0.06, 0.26)	100.00

NOTE: Weights are from random effects analysis

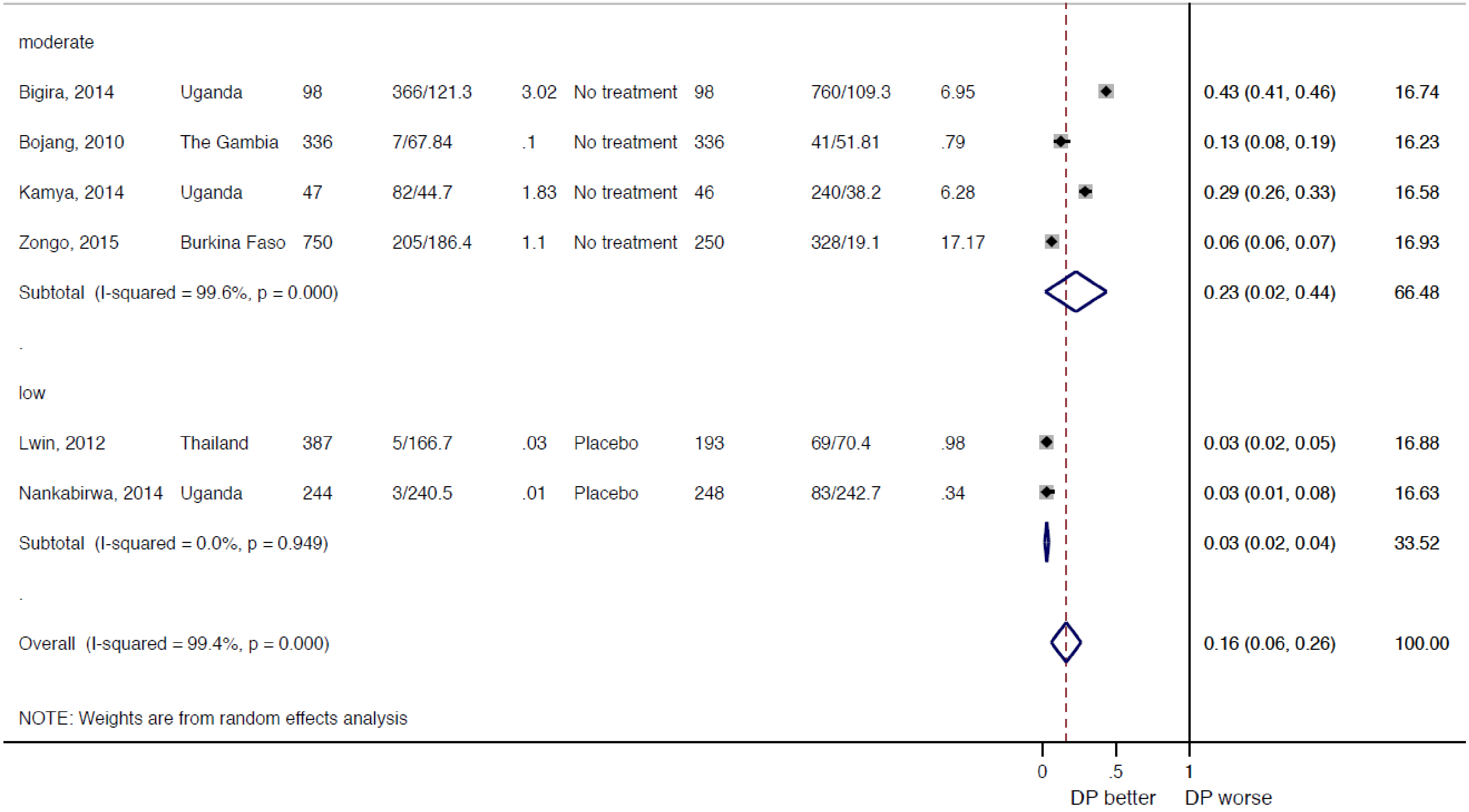

DP dihydroartemisinin-piperaquine, PYAR person years at risk, IR incidence rate, IRR incidence rate ratio
*Two studies, Lwin et al. and Zongo et al., did not report PYAR

Figure S3: Pooled incidence rate ratio for any parasitemia, monthly dihydroartemisinin-piperaquine versus placebo stratified by geography

Author, Year	Country	DP exposed	DP Events/PYAR*	DP IR	Comparator therapy	Comparator exposed	Comparator Events/PYAR	Comparator IR	IRR for any parasitemia (95% CI)	% Weight
East Africa										
Bigira, 2014	Uganda	98	366/121.3	3.02	No treatment	98	760/109.3	6.95	0.43 (0.41, 0.46)	16.74
Kamya, 2014	Uganda	47	82/44.7	1.83	No treatment	46	240/38.2	6.28	0.29 (0.26, 0.33)	16.58
Nankabirwa, 2014	Uganda	244	3/240.5	.01	Placebo	248	83/242.7	.34	0.03 (0.01, 0.08)	16.63
Subtotal (I-squared = 99.4%, p = 0.000)									0.25 (0.01, 0.49)	49.95
.										
West Africa										
Bojang, 2010	The Gambia	336	7/67.84	.1	No treatment	336	41/51.81	.79	0.13 (0.08, 0.19)	16.23
Zongo, 2015	Burkina Faso	750	205/186.4	1.1	No treatment	250	328/19.1	17.17	0.06 (0.06, 0.07)	16.93
Subtotal (I-squared = 81.7%, p = 0.019)									0.09 (0.03, 0.15)	33.16
.										
Asia										
Lwin, 2012	Thailand	387	5/166.7	.03	Placebo	193	69/70.4	.98	0.03 (0.02, 0.05)	16.88
Subtotal (I-squared = .%, p = .)									0.03 (0.02, 0.04)	16.88
.										
Overall (I-squared = 99.4%, p = 0.000)									0.16 (0.06, 0.26)	100.00

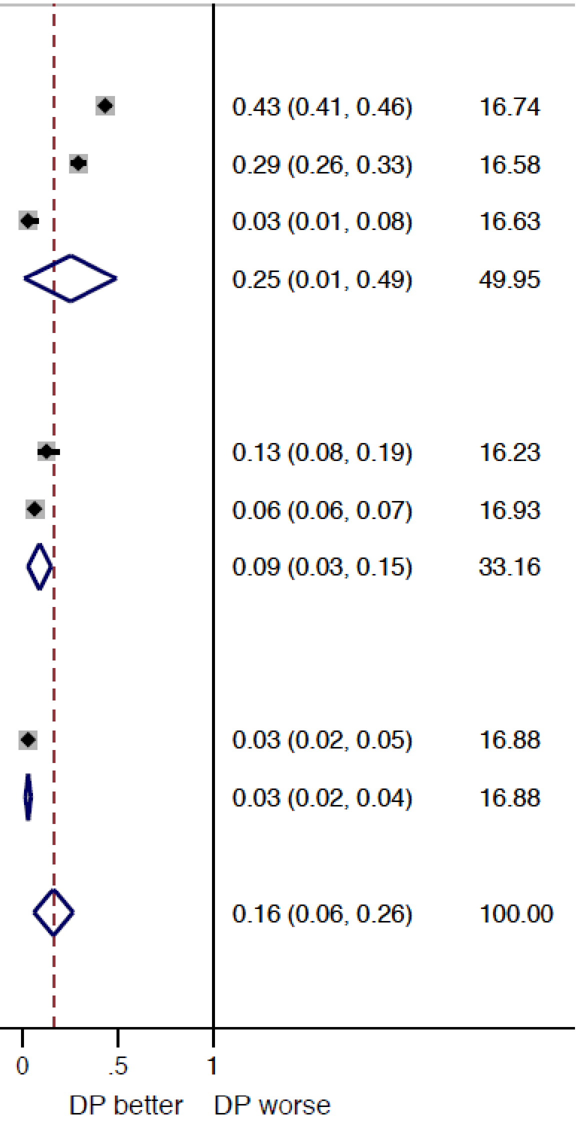

NOTE: Weights are from random effects analysis

DP dihydroartemisinin-piperaquine, PYAR person years at risk, IR incidence rate, IRR incidence rate ratio
*Two studies, Lwin et al. and Zongo et al., did not report PYAR

Figure S4: Pooled odds ratio for any hospitalization, dihydroartemisinin-piperaquine compared to other drugs and placebo

Author, Year	Country	Comparator therapy	DP: Hospitalizations/ Exposed	Comparator: Hospitalizations/ Exposed	OR (95% CI)	% Weight
Monthly DP vs. CTX for IPT						
Bigira, 2014	Uganda	CTX (d)	13/98	12/99	1.11 (0.48, 2.57)	59.74
Kamya, 2014	Uganda	CTX (d)	6/47	8/47	0.71 (0.23, 2.24)	40.26
Subtotal (I-squared = 0.0%, p = 0.543)					0.95 (0.48, 1.86)	100.00
.						
Monthy DP vs. Placebo for IPT						
Bojang, 2010	The Gambia	No treatment	3/336	8/336	0.37 (0.10, 1.40)	47.35
Kamya, 2014	Uganda	No treatment	6/47	10/46	0.53 (0.17, 1.59)	52.65
Subtotal (I-squared = 0.0%, p = 0.688)					0.45 (0.19, 1.06)	100.00
.						
Monthly DP vs. SP for IPT						
Bigira, 2014	Uganda	SP (1m)	13/98	25/98	0.45 (0.21, 0.94)	59.13
Kamya, 2014	Uganda	SP (1m)	6/47	17/46	0.25 (0.09, 0.71)	40.87
Subtotal (I-squared = 0.0%, p = 0.373)					0.37 (0.20, 0.67)	100.00
.						
Monthly DP vs. SP+PQ for IPT						
Bojang, 2010	The Gambia	SP+PQ (1m)	3/336	1/336	3.02 (0.31, 29.16)	100.00
Subtotal (I-squared = .%, p = .)					3.02 (0.31, 29.16)	100.00
.						
Monthly DP vs. SP+AQ for IPT						
Bojang, 2010	The Gambia	SP+AQ (1m)	3/336	2/336	1.50 (0.25, 9.06)	100.00
Subtotal (I-squared = .%, p = .)					1.50 (0.25, 9.06)	100.00
.						
DP vs. AL for case management						
Wanzira, 2014	Uganda	AL (3d)	13/154	42/158	0.25 (0.13, 0.50)	100.00
Subtotal (I-squared = .%, p = .)					0.25 (0.13, 0.50)	100.00
.						

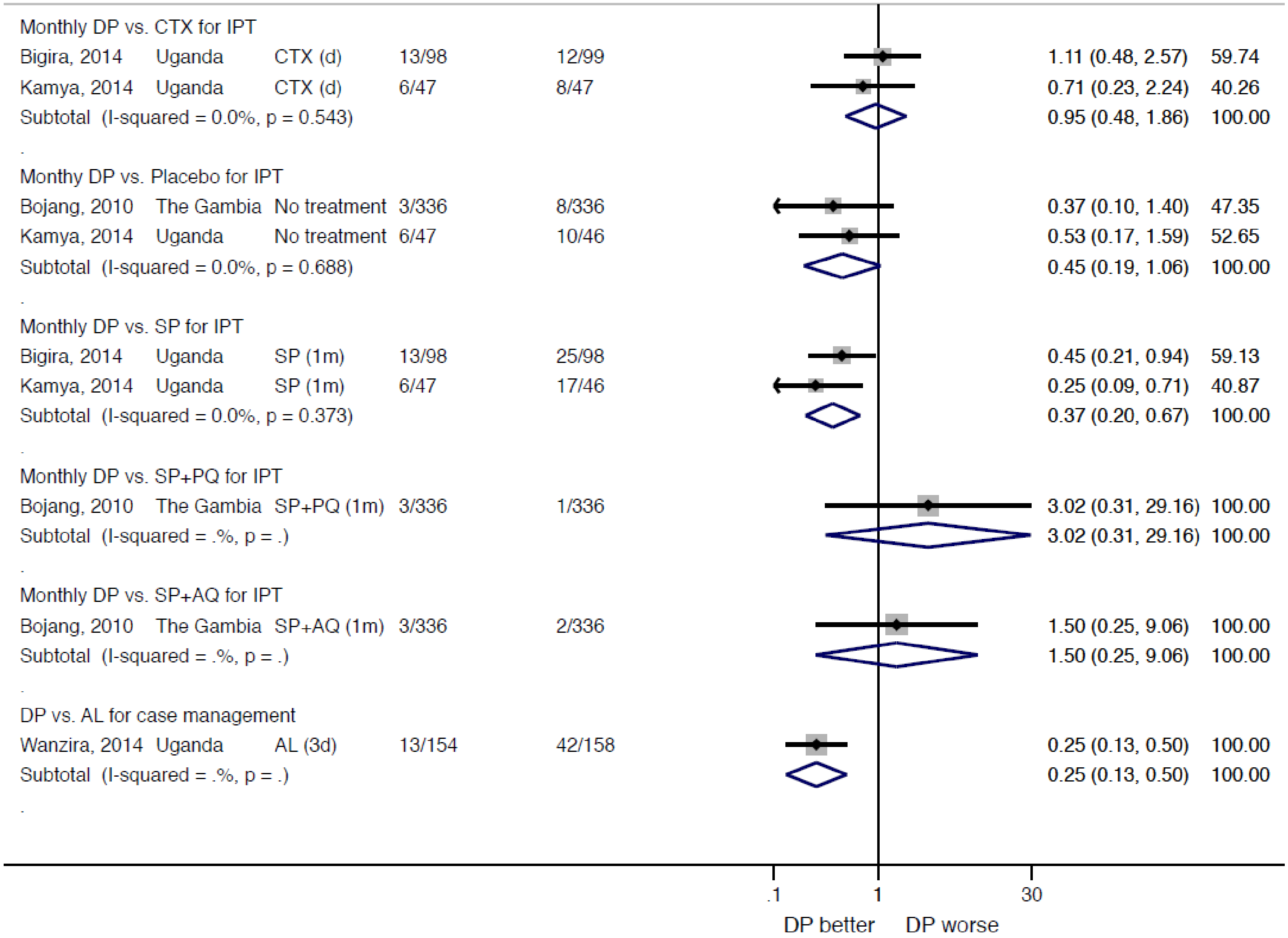

OR odds ratio, DP dihydroartemisinin-piperaquine, CTX co-trimoxazole, IPT intermittent preventative treatment, SP sulfadoxine-pyrimethamine, SP+PQ sulfadoxine-pyrimethamine piperaquine, SP+AQ sulfadoxine-pyrimethamine amodiaquine, AL artemether-lumefantrine

Figure S5: Pooled risk difference for death with dihydroartemisinin-piperaquine versus comparators

Author, Year	Country	Comparator therapy	DP: Deaths/Exposed	Comparison: Deaths/Exposed	RD (95% CI)	% Weight
**Monthly DP vs. CTX for IPT**						
Bigira, 2014	Uganda	CTX (d)	0/98	2/99	-0.02 (-0.05, 0.01)	81.64
Kamya, 2014	Uganda	CTX (d)	1/47	2/47	-0.02 (-0.09, 0.05)	18.36
Subtotal (I-squared = 0.0%, p = 0.977)					-0.02 (-0.05, 0.01)	100.00
**Monthy DP vs. Placebo for IPT**						
Bigira, 2014	Uganda	No treatment	0/98	1/98	-0.01 (-0.04, 0.02)	2.36
Bojang, 2010	The Gambia	No treatment	1/336	0/336	0.00 (-0.01, 0.01)	27.05
Kamya, 2014	Uganda	No treatment	1/47	2/46	-0.02 (-0.09, 0.05)	0.35
Lwin, 2012	Thailand	Placebo	1/387	0/193	0.00 (-0.01, 0.01)	20.48
Nankabirwa, 2014	Uganda	Placebo	0/244	0/248	0.00 (-0.01, 0.01)	29.01
Zongo, 2015*	Burkina Faso	No treatment	4/757	1/250	0.00 (-0.01, 0.01)	20.75
Subtotal (I-squared = 0.0%, p = 0.888)					0.00 (-0.00, 0.01)	100.00
**Monthly DP vs. SP for IPT**						
Bigira, 2014	Uganda	SP (1m)	0/98	2/98	-0.02 (-0.05, 0.01)	13.06
Kamya, 2014	Uganda	SP (1m)	1/47	2/46	-0.02 (-0.09, 0.05)	3.52
Desai, 2015	Kenya	SP (1m)	0/508	0/510	0.00 (-0.00, 0.00)	55.30
Kakuru, 2015	Uganda	SP (x3)	0/100	0/106	0.00 (-0.02, 0.02)	28.11
Subtotal (I-squared = 45.5%, p = 0.139)					-0.00 (-0.02, 0.01)	100.00
**Monthly DP vs. SP+PQ for IPT**						
Bojang, 2010	The Gambia	SP+PQ (1m)	1/336	0/336	0.00 (-0.01, 0.01)	36.84
Cisse, 2009	Senegal	SP+PQ (1m)	2/598	2/654	0.00 (-0.01, 0.01)	63.16
Subtotal (I-squared = 0.0%, p = 0.610)					0.00 (-0.00, 0.01)	100.00
**Monthly DP vs. SP+AQ for IPT**						
Bojang, 2010	The Gambia	SP+AQ (1m)	1/336	0/336	0.00 (-0.01, 0.01)	23.49
Cisse, 2009	Senegal	SP+AQ (1m)	2/598	2/607	0.00 (-0.01, 0.01)	37.51
Zongo, 2015*	Burkina Faso	SP+AQ (1m)	4/757	2/742	0.00 (-0.00, 0.01)	39.00
Subtotal (I-squared = 0.0%, p = 0.812)					0.00 (-0.00, 0.01)	100.00
**Monthly DP vs. Alternative DP for IPT**						
Lwin, 2012	Thailand	DP (2m)	1/387	0/381	0.00 (-0.00, 0.01)	30.45
Nankabirwa, 2014	Uganda	DP (3m)	0/244	1/248	-0.00 (-0.02, 0.01)	12.56
Desai, 2015	Kenya	IST-DP	0/508	1/510	-0.00 (-0.01, 0.00)	53.08
Kakuru, 2015	Uganda	DP (x3)	0/100	0/94	0.00 (-0.02, 0.02)	3.91
Subtotal (I-squared = 0.0%, p = 0.714)					-0.00 (-0.00, 0.00)	100.00
**DP vs. Other therapies for case management**						
Poespoprodjo, 2014	Indonesia	SP+CQ (3d)	0/408 **	0/24	0.00 (-0.05, 0.05)	0.76
Poespoprodjo, 2014	Indonesia	Quinine (7d)	0/408 **	0/402	0.00 (-0.00, 0.00)	99.24
Subtotal (I-squared = 0.0%, p = 1.000)					0.00 (-0.00, 0.00)	100.00
**DP vs. AL for case management**						
Wanzira, 2014	Uganda	AL+CTX	4/26	3/31	0.06 (-0.12, 0.23)	18.01
Wanzira, 2014	Uganda	AL (3d)	0/128	1/127	-0.01 (-0.03, 0.01)	81.99
Subtotal (I-squared = 34.0%, p = 0.218)					0.00 (-0.08, 0.09)	100.00

NOTE: Weights are from random effects analysis

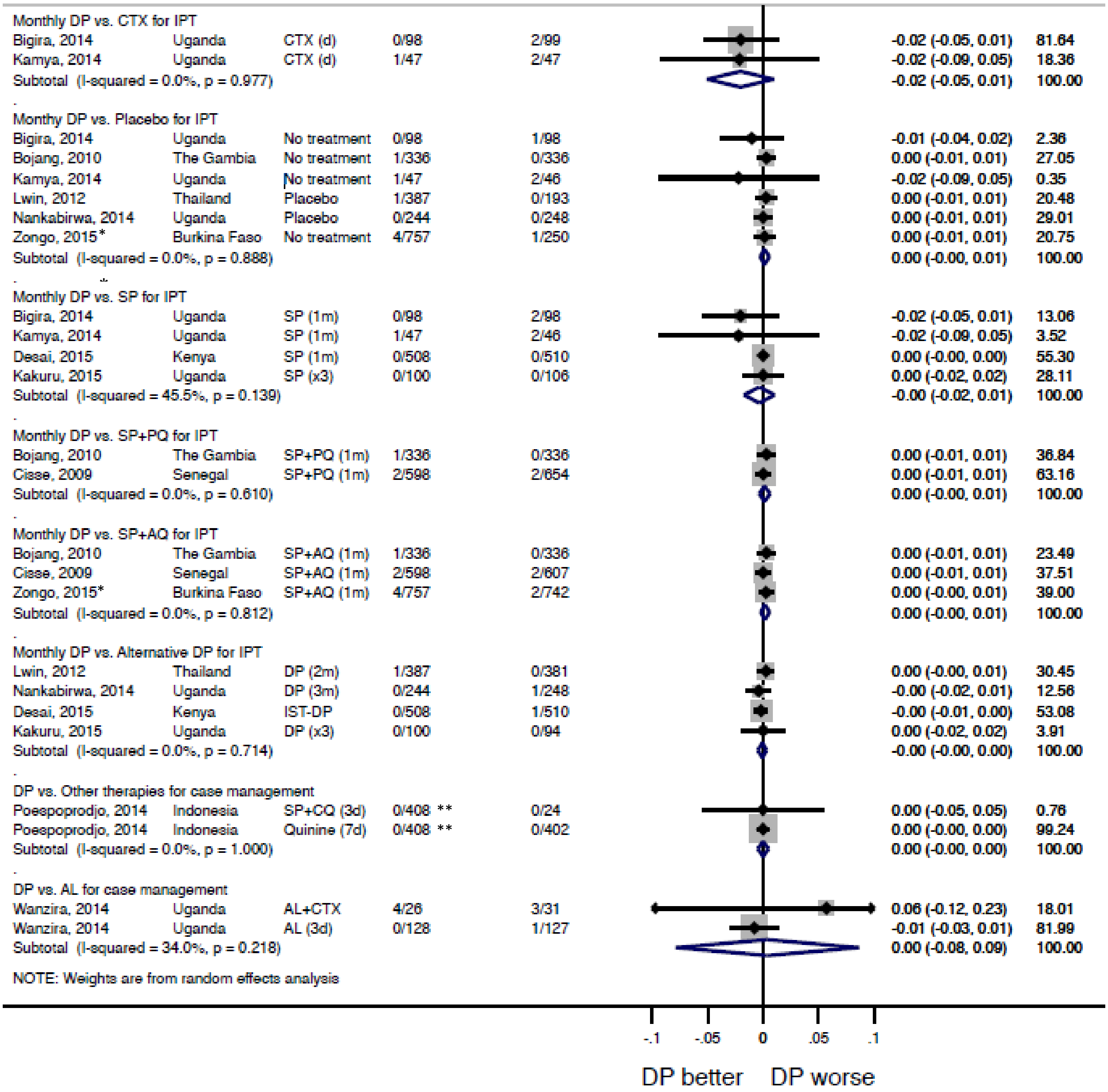

RD risk difference, DP dihydroartemisinin-piperaquine, CTX co-trimoxazole, SP sulfadoxine-pyrimethamine, SP+PQ sulfadoxine-pyrimethamine piperaquine, SP+AQ sulfadoxine-pyrimethamine amodiaquine, AL artemether-lumefantrine, IPT intermittent preventative treatment, IST intermittent screening and treatment

*Zongo et al: Numbers are based on actual drug exposures

** Poespoprodjo, et al: Only 64 of 408 DP recipients received ≥ 2 courses of DP

Figure S6: Pooled relative risk for loss to follow-up for dihydroartemisinin-piperaquine versus comparators

Author, Year	Country	Comparator therapy	DP: LTFU/Exposed	Comparison: LTFU/Exposed	RR (95% CI)	% Weight
Monthly DP vs. CTX for IPT						
Bigira, 2014	Uganda	CTX (d)	13/98	14/99	0.94 (0.47, 1.89)	84.67
Kamya, 2014	Uganda	CTX (d)	2/47	4/47	0.50 (0.10, 2.60)	15.33
Subtotal (I-squared = 0.0%, p = 0.490)					0.85 (0.45, 1.62)	100.00
.						
Monthy DP vs. Placebo for IPT						
Bigira, 2014	Uganda	No treatment	13/98	11/98	1.18 (0.56, 2.51)	11.55
Bojang, 2010	The Gambia	No treatment	7/336	11/336	0.64 (0.25, 1.62)	7.57
Kamya, 2014	Uganda	No treatment	2/47	3/46	0.65 (0.11, 3.73)	2.22
Lwin, 2012	Thailand	Placebo	135/387	48/193	1.40 (1.06, 1.86)	68.44
Nankabirwa, 2014	Uganda	Placebo	8/244	5/248	1.63 (0.54, 4.90)	5.47
Zongo, 2015*	Burkina Faso	No treatment	27/757	3/250	2.97 (0.91, 9.71)	4.76
Subtotal (I-squared = 3.1%, p = 0.397)					1.33 (1.03, 1.73)	100.00
.						
Monthly DP vs. SP for IPT						
Bigira, 2014	Uganda	SP (1m)	13/98	15/98	0.87 (0.44, 1.72)	13.36
Kamya, 2014	Uganda	SP (1m)	2/47	6/46	0.33 (0.07, 1.53)	2.73
Desai, 2015	Kenya	SP (1m)	106/516	98/515	1.08 (0.84, 1.38)	81.58
Kakuru, 2015	Uganda	SP (x3)	2/100	4/106	0.53 (0.10, 2.83)	2.33
Subtotal (I-squared = 3.3%, p = 0.376)					1.00 (0.77, 1.29)	100.00
.						
Monthly DP vs. SP+PQ for IPT						
Bojang, 2010	The Gambia	SP+PQ (1m)	7/336	3/336	2.33 (0.61, 8.95)	17.92
Cisse, 2009	Senegal	SP+PQ (1m)	47/598	50/654	1.03 (0.70, 1.51)	82.08
Subtotal (I-squared = 24.5%, p = 0.250)					1.19 (0.64, 2.21)	100.00
.						
Monthly DP vs. SP+AQ for IPT						
Bojang, 2010	The Gambia	SP+AQ (1m)	7/336	5/336	1.40 (0.45, 4.37)	17.20
Cisse, 2009	Senegal	SP+AQ (1m)	47/598	67/607	0.71 (0.50, 1.02)	47.22
Zongo, 2015*	Burkina Faso	SP+AQ (1m)	27/757	18/742	1.47 (0.82, 2.65)	35.57
Subtotal (I-squared = 59.4%, p = 0.085)					1.04 (0.59, 1.82)	100.00
.						
Monthly DP vs. Alternative DP for IPT						
Lwin, 2012	Thailand	DP (2m)	135/387	91/381	1.46 (1.17, 1.83)	40.25
Nankabirwa, 2014	Uganda	DP (3m)	8/244	14/248	0.58 (0.25, 1.36)	14.82
Desai, 2015	Kenya	IST-DP	106/516	107/515	0.99 (0.78, 1.26)	39.59
Kakuru, 2015	Uganda	DP (x3)	2/100	5/94	0.38 (0.07, 1.89)	5.35
Subtotal (I-squared = 70.6%, p = 0.017)					1.02 (0.68, 1.51)	100.00
.						
DP vs. AL for case management						
Wanzira, 2014	Uganda	AL (3d)	5/154	3/158	1.71 (0.42, 7.03)	100.00
Subtotal (I-squared = .%, p = .)					1.71 (0.42, 7.03)	100.00

NOTE: Weights are from random effects analysis

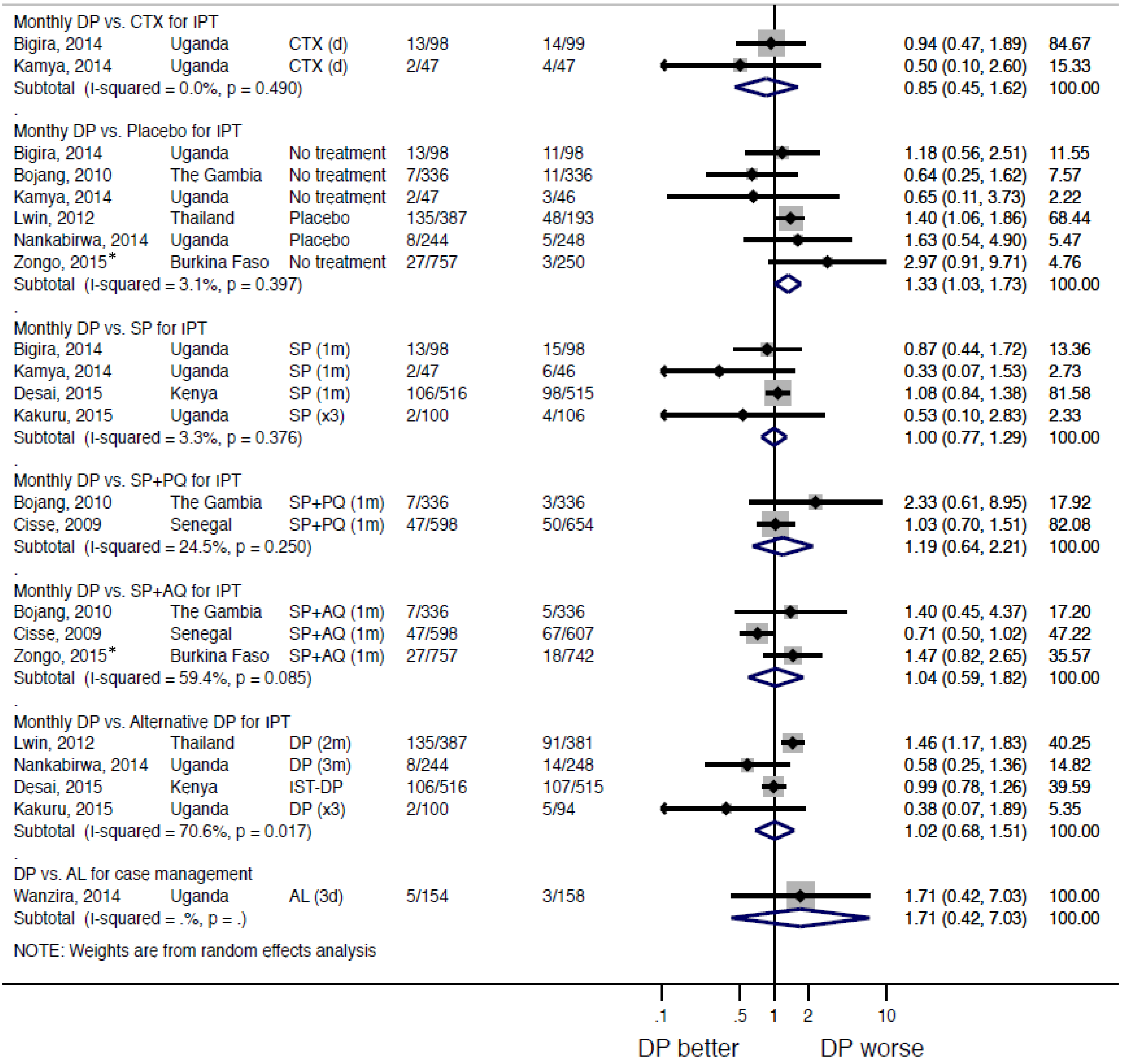

RR relative risk, DP dihydroartemisinin-piperaquine, CTX co-trimoxazole, SP sulfadoxine-pyrimethamine, SP+PQ sulfadoxine-pyrimethamine piperaquine, SP+AQ sulfadoxine-pyrimethamine amodiaquine, AL artemether-lumefantrine, IPT intermittent preventative treatment, IST intermittent screening and treatment, LTFU loss to follow-up

*Zongo et al: Numbers are based on actual drug exposures

Figure S7: Pooled relative risk for vomiting after receiving a dose of dihydroartemisinin-piperaquine compared to other drugs and placebo

Author, Year	Country	Comparator therapy	DP: Vomiting/Exposed	Comparator: Vomiting/Exposed	RR (95% CI)	% Weight
Monthy DP vs. Placebo for IPT						
Bojang, 2010	The Gambia	No treatment	17/336	38/336	0.45 (0.26, 0.78)	39.28
Lwin, 2012	Thailand	Placebo	15/387	3/193	2.49 (0.73, 8.51)	25.21
Nankabirwa, 2014	Uganda	Placebo	9/244	28/248	0.33 (0.16, 0.68)	35.51
Subtotal (I-squared = 75.3%, p = 0.017)					0.62 (0.25, 1.55)	100.00
Monthly DP vs. SP for IPT						
Desai, 2015	Kenya	SP (1m)	4/516	3/515	1.33 (0.30, 5.92)	72.09
Kakuru, 2015	Uganda	SP (x3)	1/594	2/617	0.52 (0.05, 5.71)	27.91
Subtotal (I-squared = 0.0%, p = 0.514)					1.02 (0.29, 3.63)	100.00
Monthly DP vs. SP+PQ for IPT						
Bojang, 2010	The Gambia	SP+PQ (1m)	17/336	25/336	0.68 (0.37, 1.24)	38.30
Cisse, 2009	Senegal	SP+PQ (1m)	76/598	218/654	0.38 (0.30, 0.48)	61.70
Subtotal (I-squared = 67.9%, p = 0.077)					0.48 (0.27, 0.83)	100.00
Monthly DP vs. SP+AQ for IPT						
Bojang, 2010	The Gambia	SP+AQ (1m)	17/336	19/336	0.89 (0.47, 1.69)	17.90
Cisse, 2009	Senegal	SP+AQ (1m)	76/598	76/607	1.02 (0.75, 1.37)	82.10
Subtotal (I-squared = 0.0%, p = 0.725)					0.99 (0.76, 1.30)	100.00
Monthly DP vs. Alternative DP for IPT						
Lwin, 2012	Thailand	DP (2m)	15/387	10/381	1.48 (0.67, 3.25)	35.84
Nankabirwa, 2014	Uganda	DP (3m)	9/244	24/248	0.38 (0.18, 0.80)	36.72
Desai, 2015	Kenya	IST-DP	4/516	2/515	2.00 (0.37, 10.85)	19.57
Kakuru, 2015	Uganda	DP (x3)	1/594	0/542	2.74 (0.11, 67.06)	7.87
Subtotal (I-squared = 61.6%, p = 0.050)					1.00 (0.37, 2.68)	100.00
DP vs. AL for case management						
Wanzira, 2014	Uganda	AL (3d)	80/154	167/158	0.52 (0.45, 0.61)	100.00
Subtotal (I-squared = .%, p = .)					0.52 (0.45, 0.61)	100.00

NOTE: Weights are from random effects analysis

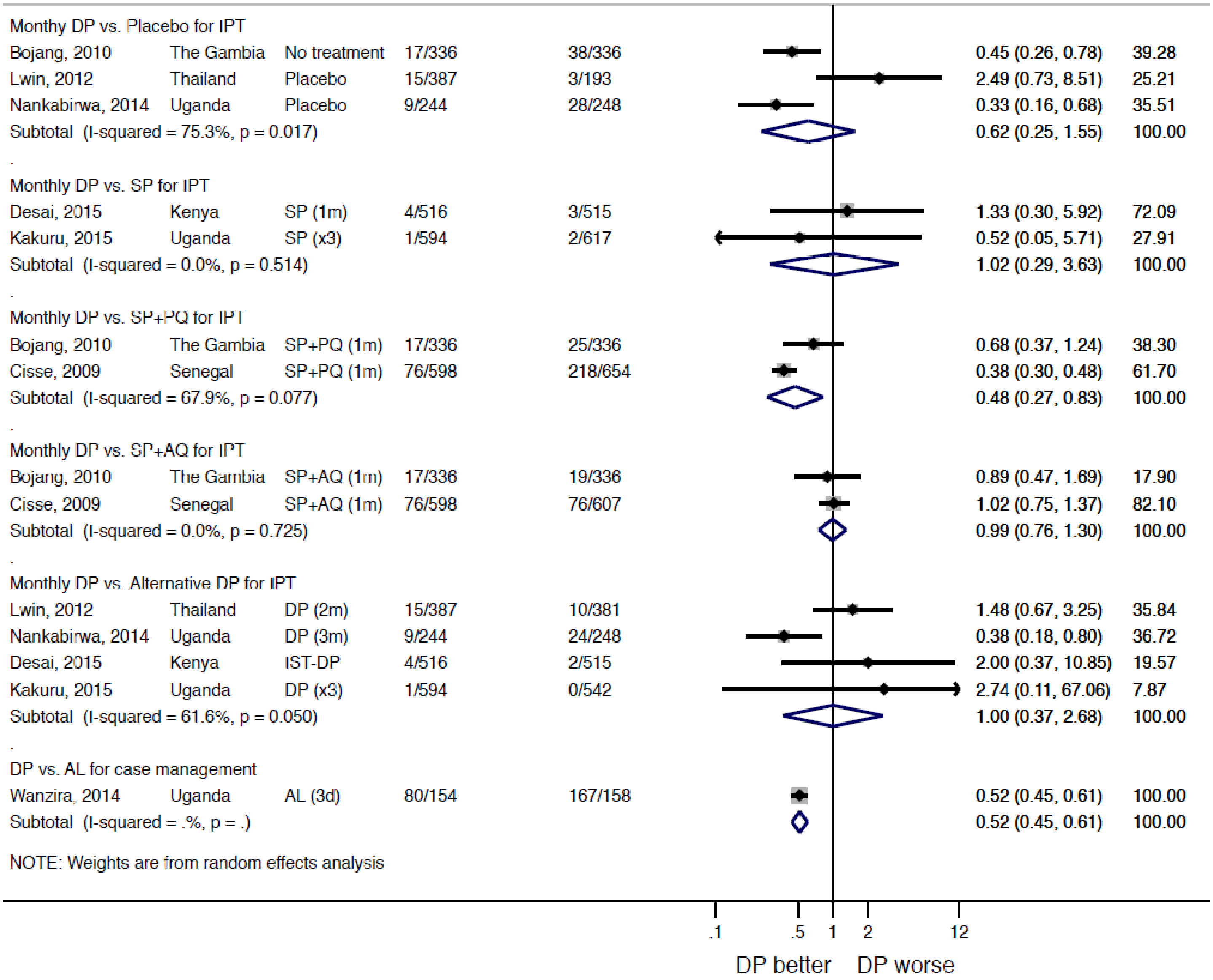

RR relative risk, DP dihydroartemisinin-piperaquine, IPT intermittent preventative treatment, SP sulfadoxine-pyrimethamine, SP+PQ sulfadoxine-pyrimethamine piperaquine, SP+AQ sulfadoxine-pyrimethamine amodiaquine, AL artemether-lumefantrine

Figure S8: Pooled relative risk for any diarrhea, dihydroartemisinin-piperaquine versus comparator therapies

Author, Year	Country	Comparator therapy	DP: Diarrhea/Exposed	Comparator: Diarrhea/Exposed	RR (95% CI)	% Weight
Monthy DP vs. Placebo for IPT						
Bojang, 2010	The Gambia	No treatment	44/336	50/336	0.88 (0.60, 1.28)	41.94
Lwin, 2012	Thailand	Placebo	20/387	5/193	1.99 (0.76, 5.23)	21.39
Nankabirwa, 2014	Uganda	Placebo	20/244	39/248	0.52 (0.31, 0.87)	36.67
Subtotal (I-squared = 68.9%, p = 0.040)					0.87 (0.48, 1.56)	100.00
.						
Monthly DP vs. SP for IPT						
Desai, 2015	Kenya	SP (1m)	1/516	0/515	1.00 (0.06, 15.91)	6.59
Kakuru, 2015	Uganda	SP (x3)	13/100	12/106	1.15 (0.55, 2.40)	93.41
Subtotal (I-squared = 0.0%, p = 0.924)					1.14 (0.56, 2.32)	100.00
.						
Monthly DP vs. SP+PQ for IPT						
Bojang, 2010	The Gambia	SP+PQ (1m)	44/336	34/336	1.29 (0.85, 1.97)	100.00
Subtotal (I-squared = .%, p = .)					1.29 (0.85, 1.97)	100.00
.						
Monthly DP vs. SP+AQ for IPT						
Bojang, 2010	The Gambia	SP+AQ (1m)	44/336	45/336	0.98 (0.66, 1.44)	100.00
Subtotal (I-squared = .%, p = .)					0.98 (0.66, 1.44)	100.00
.						

NOTE: Weights are from random effects analysis

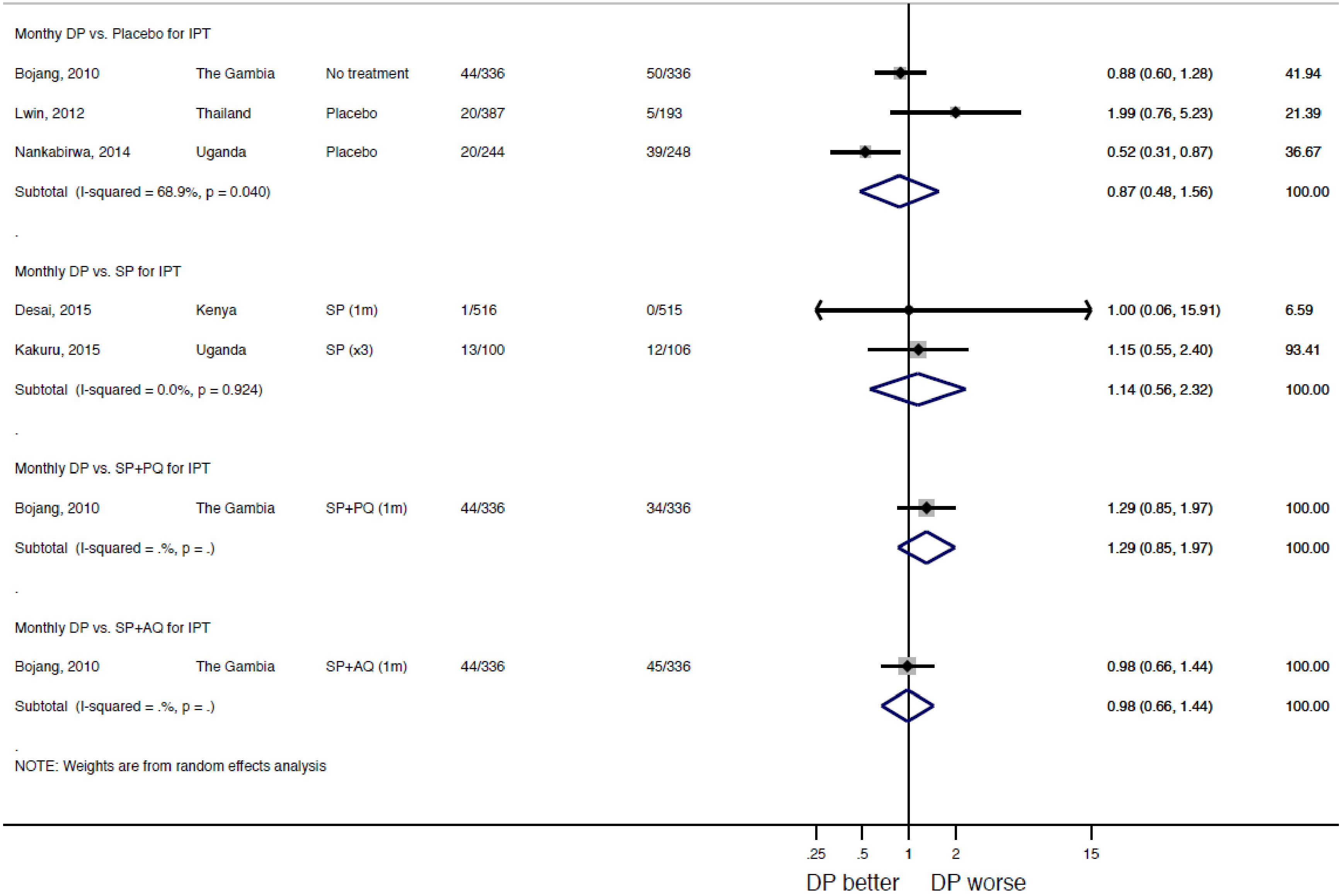

RR relative risk, DP dihydroartemisinin-piperaquine, IPT intermittent preventative treatment, SP sulfadoxine-pyrimethamine, SP+PQ sulfadoxine-pyrimethamine piperaquine, SP+AQ sulfadoxine-pyrimethamine amodiaquine

Figure S9: Pooled relative risk for any rash, dihydroartemisinin-piperaquine versus comparator therapies

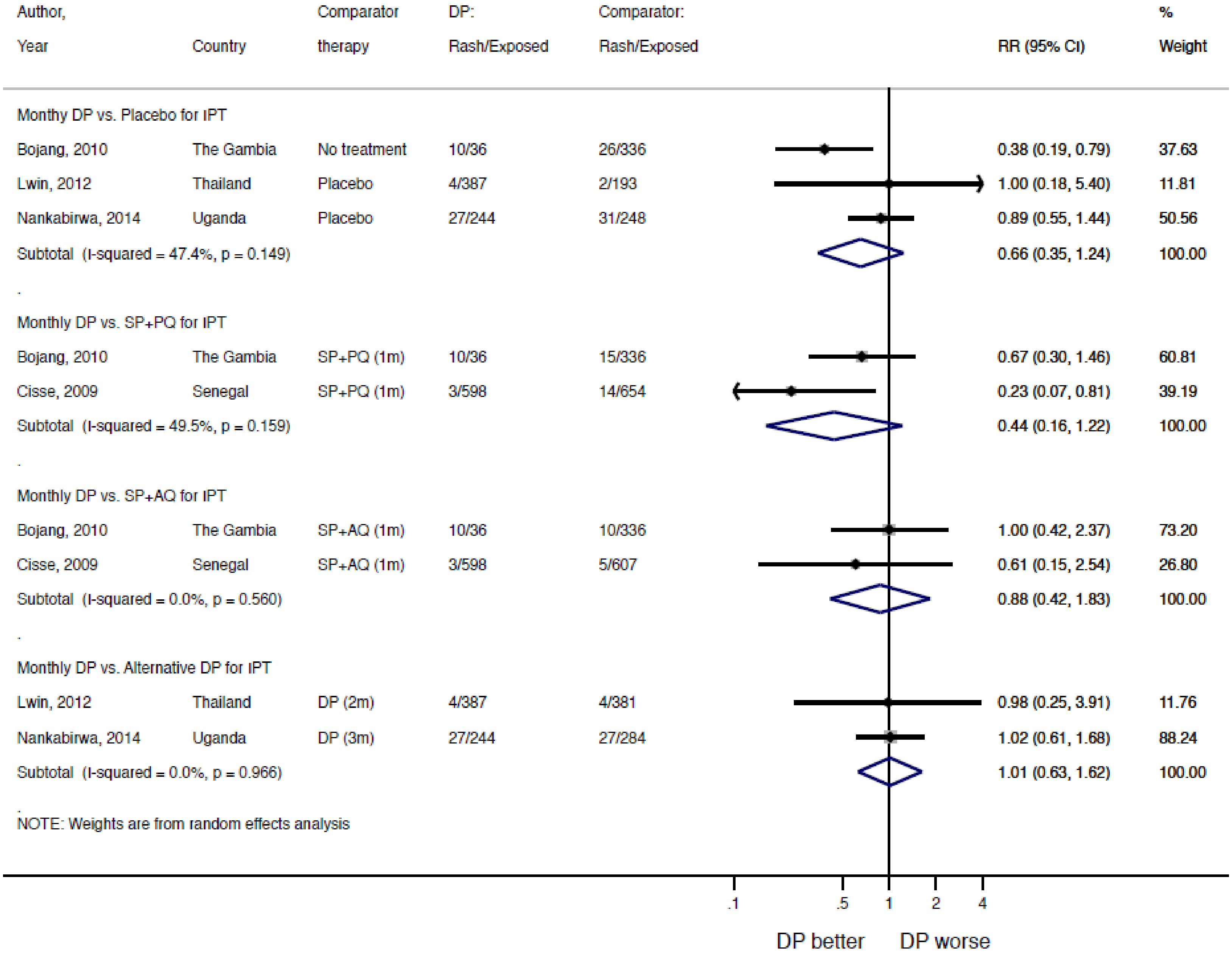

RR relative risk, DP dihydroartemisinin-piperaquine, SP+PQ sulfadoxine-pyrimethamine piperaquine, IPT intermittent preventative treatment, SP+AQ sulfadoxine-pyrimethamine amodiaquine, IST intermittent screening and treatment
